# ChatGPT and artificial hallucinations in stem cell research: assessing the accuracy of generated references – a preliminary study

**DOI:** 10.1097/MS9.0000000000001228

**Published:** 2023-09-01

**Authors:** Khan Sharun, S. Amitha Banu, Abhijit M. Pawde, Rohit Kumar, Shopnil Akash, Kuldeep Dhama, Amar Pal

**Affiliations:** a Division of Surgery; b Division of Pathology, ICAR-Indian Veterinary Research Institute, Izatnagar, Bareilly, Uttar Pradesh, India; c Department of Pharmacy, Faculty of Allied Health Science, Daffodil International University, Daffodil Smart City, Ashulia, Savar, Dhaka, Bangladesh

**Keywords:** artificial intelligence, erroneous references, fabricated references, limitations, natural language processing, reliable knowledge

## Abstract

Stem cell research has the transformative potential to revolutionize medicine. Language models like ChatGPT, which use artificial intelligence (AI) and natural language processing, generate human-like text that can aid researchers. However, it is vital to ensure the accuracy and reliability of AI-generated references. This study assesses Chat Generative Pre-Trained Transformer (ChatGPT)’s utility in stem cell research and evaluates the accuracy of its references. Of the 86 references analyzed, 15.12% were fabricated and 9.30% were erroneous. These errors were due to limitations such as no real-time internet access and reliance on preexisting data. Artificial hallucinations were also observed, where the text seems plausible but deviates from fact. Monitoring, diverse training, and expanding knowledge cut-off can help to reduce fabricated references and hallucinations. Researchers must verify references and consider the limitations of AI models. Further research is needed to enhance the accuracy of such language models. Despite these challenges, ChatGPT has the potential to be a valuable tool for stem cell research. It can help researchers to stay up-to-date on the latest developments in the field and to find relevant information.

## Introduction

Highlights

- ChatGPT has the ability to produce generally accurate references, although it was observed to occasionally generate artificial hallucinations.
- Monitoring, diverse training, and expanding knowledge cut-off can help to reduce fabricated references and hallucinations.
- Comprehending AI model strengths and limitations aids researchers in making informed decisions for integrating these technologies into stem cell research, thus advancing the field and aiding the translation of scientific discoveries into clinical applications.

Stem cell research is a dynamic and rapidly evolving field that has the potential to revolutionize medicine and improve various therapeutic applications^1^. As this field continues to advance, researchers rely on accurate and up-to-date information to support their studies and make informed decisions. With the development of artificial intelligence (AI) and natural language processing (NLP) technologies, language models like ChatGPT (Chat Generative Pre-Trained Transformer) have emerged as powerful tools for generating human-like text. These models have been trained on vast amounts of data from diverse sources (texts from the internet, books, articles, etc.), allowing them to capture the patterns and nuances of human expression^2^. This capability makes them particularly valuable for generating scientific text, including references^2^. However, the accuracy of the generated data is important for scientific integrity and the dissemination of reliable knowledge.

Furthermore, there is a concern that ChatGPT may be generating artificial hallucinations or seemingly realistic sensory experiences that do not correspond to any real-world input^3^. This could have serious implications for stem cell research, as it could lead to the use of inaccurate or misleading information. This paper aims to assess the utility of ChatGPT in stem cell research and to establish the accuracy of the references generated by the model. By evaluating the precision and reliability of ChatGPT-generated references, we will determine the feasibility of using ChatGPT as a reference-generation tool in the field of stem cell research.

## Materials and methods

### ChatGPT (Chat Generative Pre-Trained Transformer) version

The study was conducted using ChatGPT (GPT-3.5, OpenAI), which does not have access to information after September 2021 (knowledge cut-off).

### Identification of MeSH keywords

Phase one of our study involved the identification of 20 important MeSH (Medical Subject Headings) keywords in the field of stem cell research using ChatGPT with the command ‘Identify the most important 20 MeSH keywords in the field of stem cell research.’

### Generation of essays with references

Phase two involved the generation of an essay using ChatGPT with the command ‘Write an essay on “[MeSH keyword]” with references.’

For example: Write an essay on ‘Stem Cell Research’ with references.

The references generated in each of the essays were collected and manually analyzed after removing any duplicates. Only journal references were considered for analysis, and book and website references were excluded. We assessed and compared the accuracy, consistency, and appropriateness of the generated references. The references were assessed for the accurate inclusion of necessary bibliographic information, such as author names, publication titles, journal names, volume, issue, page number, and publication years.

## Results and discussion

**Figure 1:**
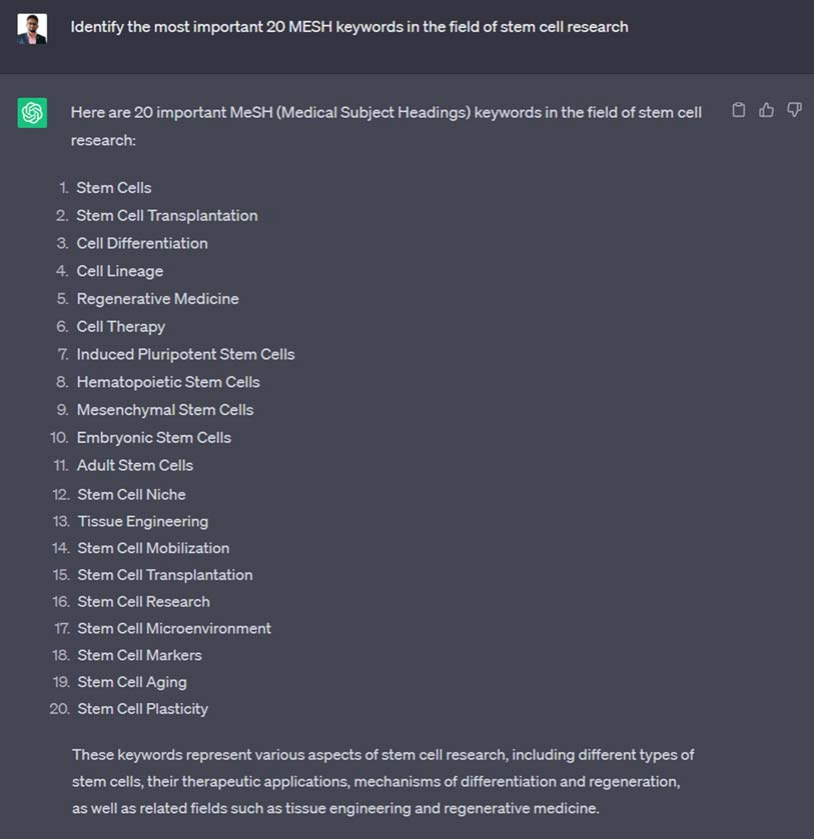
The most important 20 MeSH keywords identified by ChatGPT in the field of stem cell research.

The following MeSH keywords were identified by ChatGPT (Fig. 1): stem cells, stem cell transplantation, cell differentiation, cell lineage, regenerative medicine, cell therapy, induced pluripotent stem cells, hematopoietic stem cells, mesenchymal stem cells, embryonic stem cells, adult stem cells, stem cell niche, tissue engineering, stem cell mobilization, stem cell transplantation, stem cell research, stem cell microenvironment, stem cell markers, stem cell aging, and stem cell plasticity.

**Figure 2:**
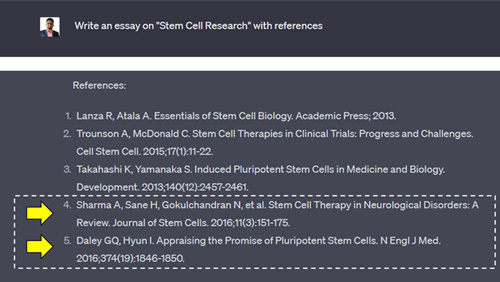
Examples of fabricated references (arrows) while generating an essay on the MeSH keyword ‘stem cell research.’

**Figure 3:**
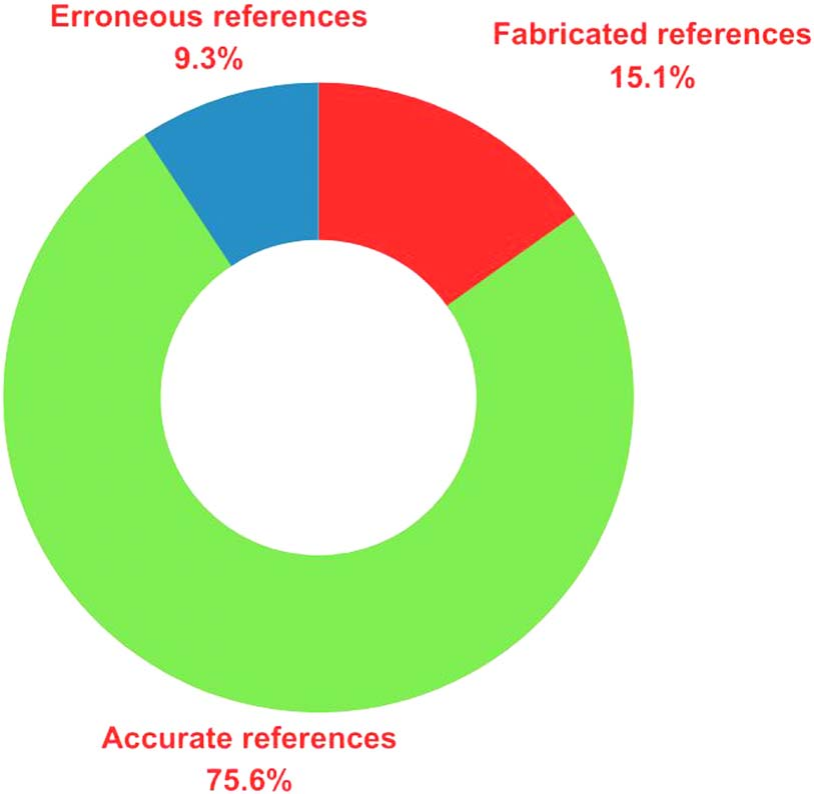
In-depth analysis of the 86 journal references linked to stem cell research. The references were divided into fabricated/fictitious references (15.12%, 13/86), erroneous references (9.30%, 8/86), and accurate references (75.58%, 65/86).

A total of 110 references were extracted from 20 essays. During the initial screening, three book references and one website reference were excluded. Furthermore, after identifying and removing 20 duplicate references, a total of 86 journal references remained for further in-depth analysis. Among the 86 journal references, 13 were identified to be fabricated/fictitious references (15.12%, 13/86) while 8 were erroneous references (9.30%, 8/86) (Figs. 2 and 3). However, the remaining 65 references were cited correctly (75.58%, 65/86). Among the eight erroneous references, five had errors in the author list, two with publication year errors, and one with article title, volume, issue, and page number errors. Similar findings were reported by Athaluri *et al*.^3^ while generating data for medical research, where 15.73% of the generated references were identified to be fabricated. On the contrary, while retrieving clinical radiological data, only 36.2% of the generated references were accurate and available through internet search, whereas the remaining 63.8% were found to be fabricated by ChatGPT^4^. The higher incidence of fabricated references can be attributed to the use of an old version of the language model (ChatGPT-3) as well as the topic used for data collection.

The higher incidence of erroneous and fabricated references in ChatGPT-generated content is attributed to the fact that it lacks real-time access to the internet or specific online databases^3^. Consequently, its responses are based on preexisting knowledge and training rather than real-time retrieval of information. Previous studies have reported artificial hallucinations while using ChatGPT for reference generation^3,5–7^. When asked for the definition of ‘artificial hallucination in language models,’ the following was the response given by ChatGPT:

*“Artificial hallucination in language models” refers to the phenomenon where language models, such as ChatGPT, generate text that mimics human-like responses but may deviate from factual or accurate information. Language models are trained on vast amounts of data and learn to generate text based on patterns and examples in the training data. However, they do not possess true understanding or knowledge. As a result, when prompted with certain queries or topics, language models like ChatGPT may generate responses that appear plausible and coherent but may not be factually correct or reliable.*

Artificial hallucination is uncommon in chatbots since they respond based on preprogrammed rules and data sets. However, in the case of advanced AI systems where new information is generated, artificial hallucination might emerge as a serious concern, especially when trained using large amounts of unsupervised data^5^. This can be resolved by training the system using a diverse and representative data set. Furthermore, continuous monitoring and detection of artificial hallucination using human evaluation or anomaly detection systems might also help in the prevention^5^. In addition, the expansion of the knowledge cut-off from September 2021 to a recent time period might also help to resolve artificial hallucination incidents while using ChatGPT^3^.

Thus, when utilizing ChatGPT-generated references, it is crucial to exercise caution and remain mindful of the possibility of fabricated references. As advised by ChatGPT itself, ‘*caution should be exercised when relying solely on the output of language models for factual or authoritative information, especially in domains where accuracy and reliability are paramount, such as scientific research or medical advice.*’ Therefore, inaccurate data generated using ChatGPT can cause harm and care must be taken while generating evidence-based expert opinions through ChatGPT^5,6^.

## Limitations

As the use of AI chatbots like ChatGPT continues to grow in the field of medical science, establishing their accuracy becomes even more imperative. Although our study is one of the preliminary studies to evaluate the accuracy of references generated using ChatGPT, the findings are limited to search queries related to stem cell research. While the results provide valuable insights into this specific domain, it is important to acknowledge that our findings are limited in scope and do not offer a comprehensive assessment of overall efficiency and accuracy. To comprehensively gauge the accuracy and efficiency of natural language processing AI chatbots like ChatGPT, additional research across diverse medical domains is necessary. Such investigations will be vital to substantiate the reliability of natural language processing AI chatbots in medical science applications.

## Conclusion and recommendations

Our results showed that ChatGPT was able to generate references that were generally accurate. However, we also found that ChatGPT sometimes generated artificial hallucinations. While language models have made significant advancements in natural language processing, they may generate references that contain errors or inaccuracies. It is crucial to assess the reliability of these references to ensure the integrity of scientific publications and prevent the propagation of misinformation. Our analysis provides insights into the strengths and limitations of ChatGPT in generating reliable references, ultimately informing researchers about the potential benefits and challenges associated with its use in stem cell research. It is worth noting that the incorporation of AI in the scientific process is an ongoing discussion. While language models like ChatGPT offer incredible potential, they are not without limitations. Contextual understanding, potential biases in training data, and the need for human oversight are aspects that require careful consideration.

Despite these challenges, ChatGPT has the potential to be a valuable tool for stem cell research. It can help researchers to stay up-to-date on the latest developments in the field and to find relevant information. Overall, the use of ChatGPT in stem cell research is a promising area of research. However, it is important to be aware of the potential risks and to take steps to mitigate them. Future research will be essential to understanding the full implications of artificial hallucinations in ChatGPT.

Researchers must remain vigilant and verify the accuracy of references before including them in their work. The rapid advancements in AI and NLP technologies offer exciting opportunities for streamlining scientific research processes. Understanding the strengths and limitations of AI models helps researchers make informed decisions about incorporating these technologies into their workflow, ultimately advancing the field of stem cell research and facilitating the translation of scientific discoveries into clinical applications. As the field of AI continues to advance, further research and development are needed to enhance the accuracy and reliability of AI-generated references.

## Ethical approval

Ethics approval was not required for this study.

## Consent

Informed consent was not required for this study.

## Sources of funding

This research did not receive any specific grant from funding agencies in the public, commercial, or not-for-profit sectors.

## Author contribution

K.S. and A.B.S.: writing original draft and conceptualization; A.M.P., R.K., S.A., K.D., and A.P.: review and editing. All authors listed have made a substantial, direct, and intellectual contribution to the work and approved it for publication.

## Conflicts of interest disclosure

The authors declare that they have no conflicts of interest.

## Research registration unique identifying number (UIN)

1. Name of the registry: not applicable.
2. Unique identifying number or registration ID: not applicable.
3. Hyperlink to your specific registration (must be publicly accessible and will be checked): not applicable.

## Guarantor

Shopnil Akash, MPharma, Research Assistant, Department of Pharmacy, Faculty of Allied Health Sciences, Daffodil International University, Dhaka 1207, Bangladesh, Tel.: +8801935567417, E-mail: shopnil29-059@diu.edu.bd; https://orcid.org/0000-0003-1751-705X.

## Data availability statement

The authors confirm that the data supporting the findings of this study are available within the article.

## Provenance and peer review

Not commissioned, internally peer-reviewed.
